# Predelivery placenta-associated biomarkers and computerized intrapartum fetal heart rate patterns

**DOI:** 10.1016/j.xagr.2022.100149

**Published:** 2022-12-16

**Authors:** Sophie Bowe, Birgitte Mitlid-Mork, Jon M. Gran, Sonia Distante, Christopher W.G. Redman, Anne Cathrine Staff, Antoniya Georgieva, Meryam Sugulle

**Affiliations:** 1 Division of Obstetrics and Gynaecology, Oslo University Hospital Ullevål, Oslo, Norway (Drs Bowe, Mitlid-Mork, Staff, and Sugulle); 2 Faculty of Medicine, University of Oslo, Oslo, Norway (Drs Bowe, Mitlid-Mork, Distante, Staff, and Sugulle); 3 Oslo Centre for Biostatistics and Epidemiology, University of Oslo and Oslo University Hospital, Oslo, Norway (Dr Gran); 4 Department of Biochemistry, Oslo University Hospital, Oslo, Norway (Dr Distante); 5 Nuffield Department of Women's & Reproductive Health, University of Oxford, Oxford, United Kingdom (Drs Redman and Georgieva).

**Keywords:** Angiogenic Factors, Cardiotocography, Computerized, Neonatal Outcome, Placental Growth Factor, Pregnancy, Soluble Fms-Like Tyrosine Kinase-1

## Abstract

**Background:**

Increasing syncytiotrophoblast stress in term and postdate placentas is reflected by increasing antiangiogenic dysregulation in the maternal circulation, with low “proangiogenic” placental growth factor concentrations and increased “antiangiogenic” soluble fms-like tyrosine kinase-1 concentrations. Imbalances in these placenta-associated proteins are associated with intrapartum fetal compromise and adverse pregnancy and delivery outcome. Cardiotocography is widely used to assess fetal well-being during labor, but it is insufficient on its own for predicting adverse neonatal outcome. Development of improved surveillance tools to detect intrapartum fetal stress are needed to prevent neonatal adverse outcome.

**Objective:**

This study aimed to assess whether predelivery circulating maternal angiogenic protein concentrations are associated with intrapartum computerized fetal heart rate patterns, as calculated by the Oxford System for computerized intrapartum monitoring (OxSys) 1.7 prototype. We hypothesized that in pregnancies with low “proangiogenic” placental growth factor levels, increased “antiangiogenic” soluble fms-like tyrosine kinase-1 levels, and increased soluble fms-like tyrosine kinase-1–placental growth factor ratio, the OxSys 1.7 prototype will generate more automated alerts, indicating fetal compromise. Our secondary objective was to investigate the relationship between maternal circulating placenta-associated biomarkers and rates of automated alerts in pregnancies with and without adverse neonatal outcome.

**Study Design:**

This was an observational prospective cohort study conducted at a single tertiary center from September 2016 to March 2020. Of 1107 singleton pregnancies (gestational week ≥37^+0^), 956 had available prelabor and predelivery placental growth factor and soluble fms-like tyrosine kinase-1 concentrations and intrapartum cardiotocography recordings. All neonatal and delivery outcomes were externally reviewed and categorized into 2 groups—the “complicated” group (n=32) and the “uncomplicated” group (n=924)—according to predefined adverse neonatal outcome. Eight different cardiotocography features were calculated by OxSys 1.7: baseline at start of cardiotocography, baseline at end of cardiotocography, short-term variation at start, short-term variation at end, nonreactive initial trace, and throughout the entire cardiotocography, maximum decelerative capacity, total number of prolonged decelerations, and OxSys 1.7 alert. OxSys 1.7 triggered an alert if the initial trace was nonreactive or if decelerative capacity and/or the number of prolonged decelerations exceeded a predefined threshold. Included women and attending clinicians were blinded to both biomarker and OxSys 1.7 results.

**Results:**

Mean maternal placental growth factor concentration was lower in the group with OxSys 1.7 alert compared with the group without the alert (151 vs 169 pg/mL; *P*=.04). There was a weak negative correlation between predelivery high soluble fms-like tyrosine kinase-1 and low short-term variation start (*r*_s_=−0.068; 95% confidence interval, −0.131 to −0.004; *P*=.036), predelivery high soluble fms-like tyrosine kinase-1 and low short-term variation end (*r*_s_=−0.068; 95% confidence interval, −0.131 to −0.005; *P*=.036), and high soluble fms-like tyrosine kinase-1–placental growth factor ratio and low short-term variation end (*r*_s_=−0.071; 95% confidence interval, −0.134 to −0.008; *P*=.027). The rate of decelerative capacity alerts increased more rapidly as placental growth factor decreased in the “complicated” compared with the “uncomplicated” group (0% to 17% vs 4% to 8%).

**Conclusion:**

More automated alerts indicative of fetal distress were generated by OxSys 1.7 in pregnancies with low maternal predelivery placental growth factor level, in line with likely increasing placental stress toward the end of the pregnancy. An antiangiogenic predelivery profile (lower placental growth factor) increased the rates of alerts more rapidly in pregnancies with adverse neonatal outcome compared with those without. We suggest that future studies developing and testing prediction tools for intrapartum fetal compromise include predelivery maternal placental growth factor measurements.

**AJOG Global Reports at a Glance**

**Why was this study conducted?**

This study was conducted to assess placental stress markers, reflected by increased antiangiogenic dysregulation in maternal circulation, and their association with objective computerized cardiotocography (CTG) measures of intrapartum fetal stress.

**Key findings**

Pregnancies with an objective computerized CTG measure of intrapartum fetal distress had a lower “proangiogenic” predelivery placental growth factor (PlGF) level, regardless of outcome. In pregnancies with adverse neonatal outcome compared with pregnancies without adverse outcome, low PlGF levels showed a trend toward more rapid increase in the rates of objective computerized CTG measures of intrapartum fetal distress.

**What does this add to what is known?**

Predelivery maternal PlGF measurement could be included in further studies testing and developing improved objective computerized CTG measures of intrapartum fetal distress.

## Introduction

The physiological processes during labor and birth challenge placental capacity and thereby fetal well-being. Timely intrapartum detection of threatening fetal compromise is decisive for neonatal outcome. Placenta-associated biomarkers, such as placental growth factor (PlGF) and soluble fms-like tyrosine kinase-1 (sFlt-1) are present in the maternal circulation during pregnancy. A low “proangiogenic” PlGF level and high “antiangiogenic” sFlt-1 level have been suggested as markers for syncytiotrophoblast stress independent of underlying clinical cause, thus representing general “placental health markers.”^1^^,^^2^ Imbalances in circulating PlGF and sFlt-1 have been shown to be promising predictors of intrapartum fetal compromise at term^3^, ^4^, ^5^ and adverse pregnancy and delivery outcome in postdate pregnancies.^6^

Established intrapartum tools for surveying fetal well-being include conventional cardiotocography (CTG), optional addition of fetal scalp blood lactate or pH measurement in situations of nonreassuring CTG tracings, and ST waveform analysis (STAN) of the fetal electrocardiogram.^7^ Both fetal scalp blood analysis and STAN require broken membranes and fetal scalp manipulation.^8^ A major limitation of CTG analysis is the subjective interpretation with poor interobserver agreement^9^ and fair to good intraobserver agreement.^9^^,^^10^ Thus, there is a lack of optimal intrapartum tools to identify term births that are most at risk of severe fetal complications.

Computerized electronic CTG analysis aims to increase the sensitivity without increasing the false-positive rate of detecting severe compromise. With the use of large sets of routinely collected CTG and maternity data, Georgieva et al^11^ have developed a computerized data-driven prototype for electronic fetal heart rate (FHR) monitoring evaluation: the Oxford System for computerized intrapartum monitoring, OxSys. This research tool is trained to generate automated alerts when a fetus is at risk of intrapartum hypoxia. Its offline application on retrospective data demonstrated that OxSys 1.5 is comparable to clinical judgment.^11^ The system is based on novel nonclassical and standard classical CTG characteristics, with the addition of clinical risk factors, and is currently being researched, developed, and further updated.^11^

Our primary study aim was to assess whether imbalances in predelivery maternal placenta-associated protein concentrations, representing markers of placental health, are associated with OxSys 1.7 computerized FHR characteristics intrapartum, at term, and beyond term, regardless of clinical outcome groups. We hypothesized that in term and postdate pregnancies with impaired placental health (as evaluated by low maternal circulating “proangiogenic” PlGF level, increased “antiangiogenic” sFlt-1 level, and increased sFlt-1–PlGF ratio before delivery), the OxSys 1.7 prototype will generate more automated alerts. Our secondary objective was to investigate the relationship between circulating PlGF and sFlt-1 and rates of automated alerts in pregnancies with and without adverse neonatal outcome.

## Materials and Methods

### Study design and participants

**Fig. 1:**
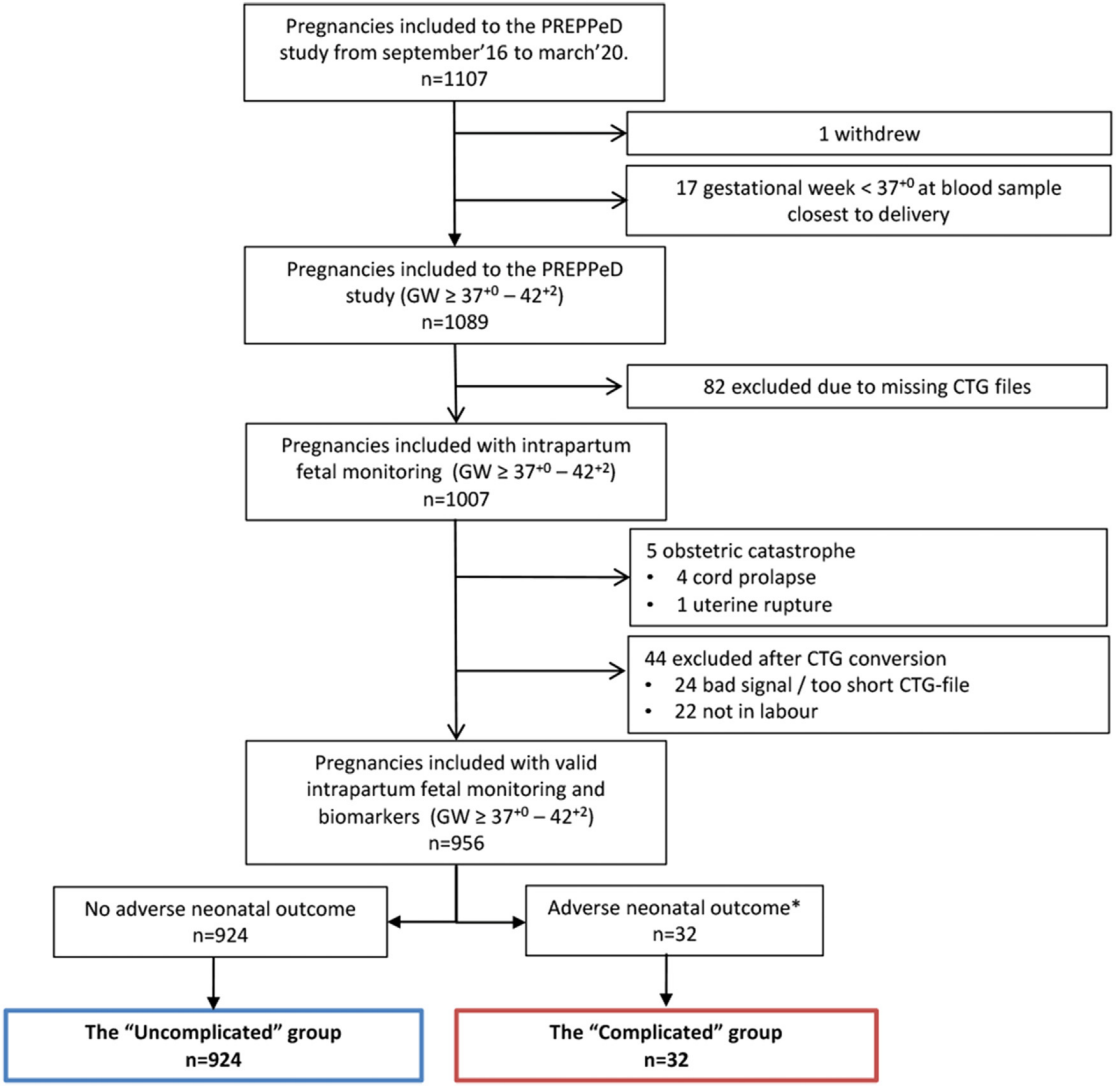
Flow chart of the final study cohort (n=956) with valid intrapartum fetal monitoring and biomarkers (GW ≥37+0–42+2) Asterisk indicates as defined in Table 1. *CTG*, cardiotocography; *GW*, gestational week; *PREPPeD*, PREdelivery Placental biomarkers– Pregnancy and Delivery outcome. *Bowe. Placenta-associated biomarkers and objective cardiotocography patterns. Am J Obstet Gynecol Glob Rep 2022.*

This study is part of our ongoing prospective PREPPeD (PREdelivery Placental biomarkers – Pregnancy and Delivery outcome) study^6^^,^^12^ at the Department of Obstetrics, Oslo University Hospital (OUH), Ullevål, delivering approximately 7000 women annually. The PREPPeD study is investigating whether maternal blood biomarkers in late pregnancy correlate with delivery outcomes in both healthy and complicated pregnancies. Women with singleton pregnancy at and beyond term (gestational week [GW] ≥37^+0^) referred for any clinical question requiring specialized consultation, such as reduced fetal movements, diagnosed or suspected preeclampsia (or other pregnancy-induced hypertensive disorder) and/or suspected fetal growth restriction, diabetes mellitus, and/or routine clinical postdate evaluation or planning of induction of labor, were included from September 2016 to March 2020 (Figure 1).

Exclusion criteria were: non-Norwegian or non-English language, communicable disease, and age <18 years. Gestational age and estimated date of delivery (40^+2^) were calculated on the basis of routine ultrasound screening at GW 17 to 20 according to Norwegian national guidelines, or when not available, from last menstrual period. Birthweight percentile was calculated according to Norwegian population-based sex-adjusted reference ranges.^14^ Small for gestational age was defined as a birthweight <10th sex-adjusted Norwegian percentile.^14^

Women and clinicians were blinded to biomarker and OxSys 1.7 results. Labor and delivery were managed according to Department protocol. Clinical diagnosis of intrapartum fetal distress was made by the attending obstetrician on the basis of abnormal FHR patterns and/or high fetal scalp lactate >4.8 mmol/L. Validation of umbilical cord blood gases has been described in previous publications^6^ and was based on Kro et al.^15^

Recruited women gave informed written consent. National research ethical and institutional bodies have approved the PREPPeD study, which this study is part of (Regional Committee for Medical and Health Research Ethics [REK] South East Norway: ref 2016/652; approval date: May 20, 2016). The PREPPeD biobank is coordinated as a thematic biobank within the Oslo Pregnancy Biobank (REK Eastern Norway: ref 529-02162; approval date: December 13, 2002). The PREPPeD study is registered at ClinicalTrials.gov (NCT0310008).

### Study sampling and procedures

A venous maternal blood sample was taken at study inclusion and if possible daily until labor onset. Further details of storage and centrifugation of the blood samples have been described previously.^6^^,^^12^ In women with repeated predelivery samples, maternal serum PlGF and sFlt-1 were analyzed from the blood sample closest to labor onset. The maternal serum PlGF and sFlt-1 concentrations were analyzed postpartum, and sFlt-1–PlGF ratio derived, blinded for clinical information, at the Department of Medical Biochemistry, OUH, on cobas e 801 and cobas e 602 analyzers (F. Hoffmann-La Roche AG, Basel, Switzerland). The PlGF and sFlt-1 concentrations were quantified using the fully automated Elecsys system (Roche Diagnostics, Rotkreuz, Switzerland), according to the manufacturer´s instructions, and all concentrations were within the detection ranges (PlGF: 3–10,000 pg/mL and sFlt-1: 10–85,000 pg/mL, respectively). The coefficients of variation were ≤3.7% for PlGF and ≤1.7% for sFlt-1.

### The Oxford System for computerized electronic intrapartum monitoring

All available CTG recordings from labor were acquired with a STAN S31 (Neoventa Medical, Moelndal, Sweden) and were later decoded by the manufacturer (Neoventa Medical AB) blinded for clinical information to extract the CTG data. Conventional, visual CTG interpretation was done by the clinicians during labor and delivery blinded for biomarker results, and the ST analysis of fetal electrocardiogram was not on screen and was not part of the clinical assessment. The full CTG recordings for each woman were analyzed postpartum with OxSys 1.7. The Oxford 1.5 system is described in detail elsewhere.^11^ In this study, the OxSys 1.7 prototype was used, based on OxSys 1.5,^11^^,^^16^^,^^17^ but with updates on the algorithms for noise removal, parameters of decelerative capacity (DC) calculations, and prolonged decelerations. The DC of the phase-rectified signal averaging algorithm is the combined measurement of depth, frequency, and slope of any dips in heart rate,^11^^,^^16^^,^^18^ measuring the frequency and magnitude of FHR decelerations.^17^ In this study, OxSys 1.7 was calculated as:

- Baseline FHR at the onset of recording and at its end, measured in beats per minute (bpm) during the first 60 minutes and last 60 minutes of the CTG trace; the longest duration available if <60 minutes
- Short-term variation (STV) at the start of recording and at its end, measured in milliseconds during the first 30 minutes and last 30 minutes of the CTG trace
- Maximum DC during the entire CTG trace
- Total number of prolonged decelerations (≥3 minutes) for the entire CTG trace
- Nonreactive initial trace (first 60 minutes, yes/no)
- Activation of the OxSys 1.7 alert at any point (yes/no)
  o OxSys 1.7 alerted if at any point, one of the following conditions were met:
    • Nonreactive initial trace^11^
    • Thick meconium and DC >5.0 bpm
    • Preeclampsia and DC >6 bpm
    • DC >6.7 bpm regardless of clinical risk factors
    • Think meconium and prolonged decelerations

CTG recordings ending at ≥3 hours before delivery and CTG traces <15 minutes were excluded.

### Assignment of pregnancies to the “complicated” and “uncomplicated” group

All neonatal and delivery outcomes were externally reviewed by a “Diagnostic Advisory Group” (DAG),^6^^,^^12^ consisting of 2 senior consultant obstetricians not affiliated with the study and blinded for biomarker and OxSys 1.7 results. After a postpartum review of the mother's and neonate's medical records (including placental histology, where available), the DAG concluded whether there was a predefined adverse neonatal outcome (Table 1). In case of dissent, a third senior consultant obstetrician, equally independent and blinded for biomarker and OxSys 1.7 results, reviewed and adjudicated the case. If a pregnancy resulted in an adverse neonatal outcome, it was included in the “complicated” group (Figure 1). If there was no adverse neonatal outcome, the respective pregnancy was included in the “uncomplicated” group (Figure 1).

**Table 1:** Primary adverse neonatal outcomes as defined for this study

A: Primary adverse outcomes (Either of the composite adverse outcomes 1–8):	
1	Fetal acidemia, evaluated by: A. Arterial umbilical cord blood gases (pH, base deficit [BD]): in neonates from labored delivery (regardless of subsequent method, vaginal or cesarean delivery): umbilical artery blood pH <7.05 and arterial BD >14 OR B. Umbilical artery blood lactate above reference level for respective gestational age^13^
2	Newborn low Apgar A. <4 at 1 min OR B. <7 at 5 min (any newborn intubated at this time point will be registered as low Apgar at 5 min because Apgar cannot be assessed in assisted ventilation)
3	Newborn asphyxia: defined as fetal acidemia (#1 above) AND newborn low Apgar (#2 above)
4	Rate of intrauterine fetal demise/intra-/postpartum fetal death
5	Neonatal intubation/mechanical ventilation >6 h
6	Meconium aspiration syndrome
7	Neonatal hypoxic-ischemic encephalopathy
8	Therapeutic hypothermia of the neonate

Bowe. Placenta-associated biomarkers and objective cardiotocography patterns. Am J Obstet Gynecol Glob Rep 2022.

### Statistical analysis

Statistical analyses were performed using IBM SPSS Statistics for Windows, Version 25.0 (IBM Corp, Armonk, NY), Stata Special Edition, Version 16.1 (StataCorp LLC, College Station, TX), and MATLAB and Statistics Toolbox, Release R2020b (MathWorks, Inc, Natick, MA).

Clinical baseline characteristics of the participants are presented by descriptive statistics. Categorical variables are expressed as number and percentage, and the 2 groups were compared using the Fisher exact test. Continuous data in Table 2 are expressed as medians and interquartile ranges, and the 2 groups were compared by Kruskal–Wallis test. Biomarker measurements were right-skewed, and therefore log-transformed. Means were compared with independent sample *t*-test and 1-way analysis of variance, and subsequent pairwise comparisons between groups were Bonferroni-corrected. Means presented in the Results section were obtained by back transformation using the exponential function. The correlation between the biomarkers and OxSys parameters was analyzed with the Spearman correlation coefficient.

**Table 2:** Clinical characteristics of the pregnancy cohort with intrapartum fetal monitoring (cardiotocography cohort) (gestational weeks 37^+0^–42^+2^; n=956) in the “uncomplicated” group without adverse outcome and the “complicated” group with adverse neonatal outcome (Table 1)

Characteristics	“Uncomplicated” n=924	“Complicated” n=32	*P* value
Nulliparous, n (%)	587 (63.5)	24 (75.0)	.261^a^
Maternal age in years at inclusion, median (IQR)	33.0 (30.0–36.0)	33.5 (31.3–37.5)	.157^b^
Body mass index at first trimester,^c^ median (IQR)	23.0 (21.2–25.5)	23.3 (21.7–25.1)	.593^b^
Systemic blood pressure at inclusion,^c^ median (IQR)	122 (117–130)	120 (115–134)	.497^b^
Diastolic blood pressure at inclusion, median (IQR)	79 (72–85)	78 (73–84)	.811^b^
Diabetes mellitus (GDM, DM), n (%)	62 (6.7)	3 (9.4)	.474^a^
Serum creatinine at inclusion (µmol/L),^c^ median (IQR)	54.0 (49.0–61.0)	55.0 (49.3–60.0)	.888^b^
Hypertensive pregnancy disorder, n (%)	139 (15.0)	4 (12.5)	>.999^a^
Maternal smoking/snus (moist tobacco), n (%)	150 (16.2)	4 (12.5)	.806^a^
Ethnicity, n (%)			.093^a^
White	841 (91.0)	26 (81.3)	
Black or Afro-American	37 (4.0)	2 (6.3)	
Asian	37 (4.0)	4 (12.5)	
Other	9 (1.0)	0	
Completed educational level,^c^ n (%)			.448^a^
Primary school	10 (1.1)	1 (3.1)	
High school	116 (12.6)	3 (9.4)	
University/college <4 y	293 (31.7)	12 (37.5)	
University/college >4 y	503 (54.4)	16 (50.0)	
Gestational age at blood sample closest to delivery in days, median (IQR)	286 (276–291)	286 (282–291)	.250^b^
Gestational age at delivery in days, median (IQR)	289 (279–293)	290 (284–294)	.189^b^
Days from blood sample to delivery in days, median (IQR)	1.0 (1.0–3.0)	1.0 (1.0–3.0)	.718^b^
Child male sex, n (%)	504 (54.5)	17 (53.1)	>.999^a^
Birthweight, median (IQR)	3670 (3306–3994)	3838 (3400–4354)	.039^b^
Small for gestational age, n (%)	132 (14.3)	4 (12.5)	>.999^a^
Deliveries (total), n (%)			<.001^a^
Vaginal (nonoperative)	636 (68.8)	10 (31.3)	
Vacuum/forceps	150 (16.2)	11 (34.4)^d^	
CD	138 (14.9)	11 (34.4)^e^	
Induction of labor, n (%)	674 (72.9)	23 (71.9)	.842^a^
Total number of days the neonate was admitted to NICU, median (IQR)	3.0 (1.0–4.3)	3.5 (1.3–8.8)	.423^b^

*CD*, cesarean delivery; *DM*, diabetes mellitus; *GDM*, gestational diabetes mellitus; *IQR*, interquartile range; *NICU*, neonatal intensive care unit.

a Fisher exact test

b Kruskal–Wallis test

c Missing data: body mass index at first trimester for 2 in “uncomplicated” group, systolic and diastolic blood pressure at inclusion for 2 in “uncomplicated” group, serum creatinine for 27 in “uncomplicated” group, educational level at study inclusion for 2 in “uncomplicated” group

d Indications for operative vaginal delivery in the complicated group were fetal distress alone (n=5), prolonged second stage of labor alone (n=2), and the remaining (n=4) were a combination of fetal distress and prolonged second stage of labor

e Indications for acute cesarean delivery in the complicated group were fetal distress (n=5), prolonged second stage of labor (n=1), cephalopelivic disproportion (n=1), malpresentation (n=1), fetal distress and malpresentation (n=1), and preeclampsia and prolonged first stage of labor (n=1). *Bowe. Placenta-associated biomarkers and objective cardiotocography patterns. Am J Obstet Gynecol Glob Rep 2022.*

Event Rate Estimate (EveREst) plots^19^ were used to display how maternal circulating PlGF levels relate to DC >6.7 bpm, that is, PlGF values were grouped into quantiles, with each of them containing 20% of the deliveries. The minimal PlGF values for each quantile group are shown on the horizontal axis. The quantiles were plotted against percentage rates (event rates) for a DC >6.7 bpm on the vertical axis. Proportions were compared with the chi-square test.

## Results

In total, 1107 women (GW ≥37^+0^) were recruited before delivery. The data from 956 pregnancies (CTG cohort) were analyzed and categorized into an “uncomplicated” group of 924 women with no adverse neonatal outcome and a “complicated” group of 32 women with adverse neonatal outcome (Figure 1). The distribution of the adverse neonatal outcome components for the “complicated” group (n=32) is shown in Supplemental Table 1. When comparing the clinical characteristics between the 2 groups (Table 2), the rate of operative deliveries (vaginal and acute cesarean) was higher in the “complicated” than in the “uncomplicated” group (*P*<.001) (Table 2). The median gestational age at blood sample closest to delivery was similar for the 2 groups (286 days).

### Maternal circulating biomarkers and correlation with OxSys 1.7 parameters

**Fig. 2:**
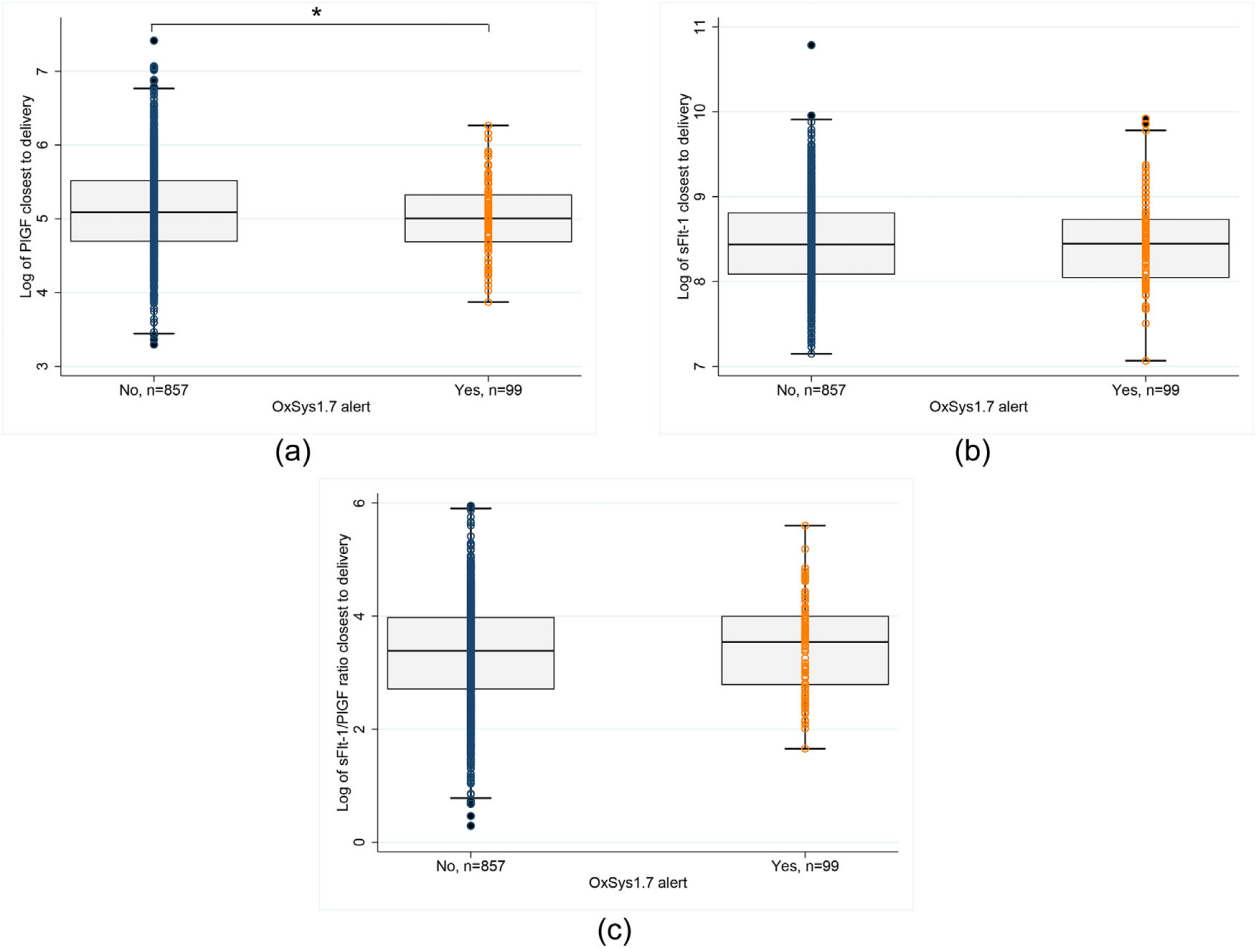
Logarithmic scale values of maternal serum PlGF level, serum sFlt-1 level, and sFlt-1–PlGF ratio for the pregnancy cohort with intrapartum fetal monitoring (CTG cohort) (GW 37^+0^–42^+2^; n= 956) A, PlGF; B, sFlt-1; C, sFlt-1–PlGF ratio. The *large horizontal bar* shows the median value for the OxSys 1.7 alert (yes/no), and the *smaller bars* show the interquartile ranges. *Asterisk* indicates significant on a 0.05 level. *CTG*, cardiotocography; *GW*, gestational week; *PlGF*, placental growth factor; *s*Flt-1, soluble fms-like tyrosine kinase-1. *Bowe. Placenta-associated biomarkers and objective cardiotocography patterns. Am J Obstet Gynecol Glob Rep 2022*.

Logarithmic values of the mean maternal PlGF concentration from blood sample closest to delivery are shown in Figure 2 for the groups with and without OxSys 1.7 alert. Mean predelivery maternal PlGF concentration was significantly lower in the group of pregnancies that subsequently had OxSys 1.7 alert intrapartum when compared with that of the group without OxSys 1.7 alert (151 vs 169 pg/mL; *P*=.04) (Figure 2, A). The mean sFlt-1 concentration and sFlt-1–PlGF ratio were higher when there was an OxSys 1.7 alert compared with no OxSys 1.7 alert, but the difference was not significant (Figure 2, B and C).

**Fig. 3:**
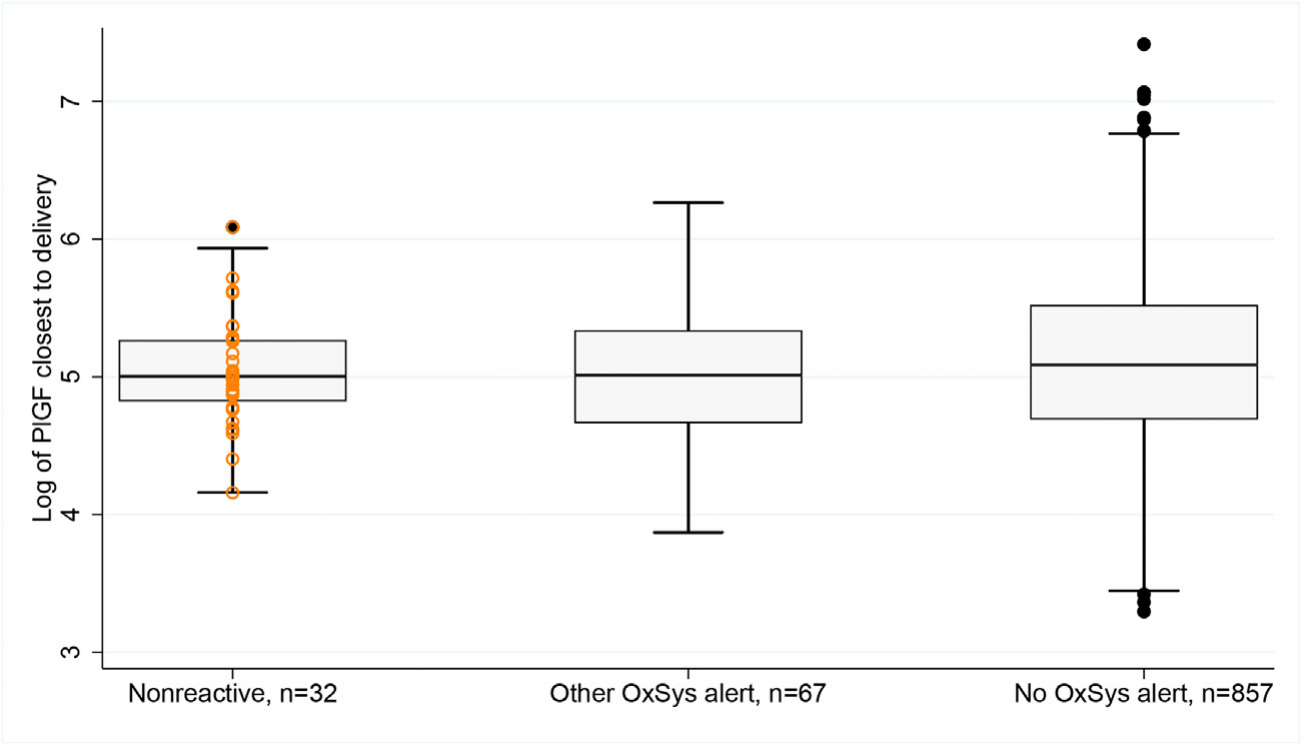
Logarithmic scale values of maternal serum PlGF level for the pregnancy cohort with intrapartum fetal monitoring (CTG cohort) (GW 37^+0^–42^+2^; n= 956) divided into nonreactive cases (n=32), other OxSys alert (n=67), and no OxSys alert (n=857) *CTG*, cardiotocography; *GW*, gestational week; *PlGF*, placental growth factor.*Bowe. Placenta-associated biomarkers and objective cardiotocography patterns. Am J Obstet Gynecol Glob Rep 2022*.

When comparing the mean maternal PlGF concentration for the groups with a “nonreactive alert,” “other OxSys alert,” and “no OxSys alert” (Figure 3), the PlGF level was highest when there was no OxSys alert, but the difference was not significant among the 3 groups (155 vs 150 vs 169 pg/mL; *P*=.23). No significant difference was found when comparing the mean sFlt-1 level and sFlt-1–PlGF ratio among the 3 alert groups (not shown).

The correlation between the placenta-associated biomarkers PlGF, sFlt-1, and sFlt-1–PlGF ratio and the other CTG parameters from OxSys 1.7 (maximum DC, baseline initial, baseline end, STV start, and STV end) was analyzed for the CTG cohort (n=956). A weak negative correlation was found between high sFlt-1 and low STV start (*r*_s_=−0.07; 95% confidence interval [CI], −0.131 to −0.004; *P*=.04), high sFlt-1 and low STV end (*r*_s_=−0.07; 95% CI, −0.131 to −0.005; *P*=.04), and high sFlt-1–PlGF ratio and low STV end (*r*_s_=−0.07; 95% CI, −0.134 to −0.008; *P*=.03) (Supplemental Table 2).

### EveREst plots

**Fig. 4:**
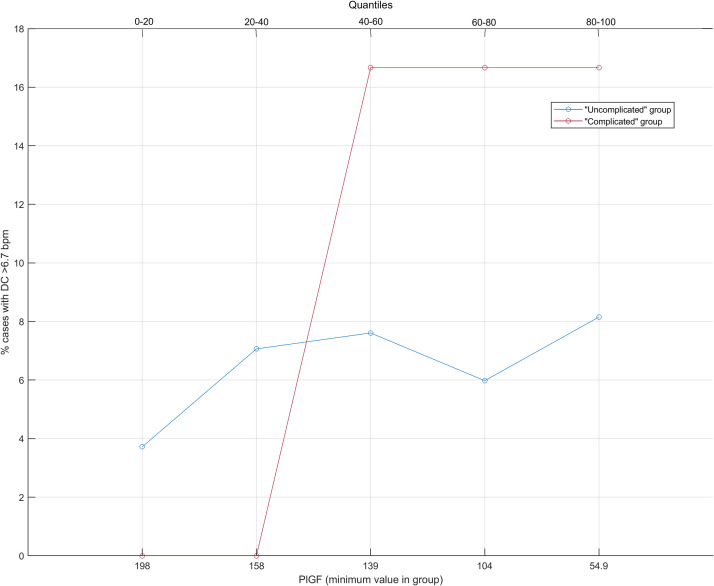
EveREst plots showing the relationship of PlGF value to cases with DC >6.7 bpm in the uncomplicated (*blue*) and the complicated (*red*) group Each point contains 20% of the population. The event rate or positive predictive values are plotted on the vertical axis. The minimum values for PlGF for each 20th quantile are on the horizontal axis. *bpm*, beats per minute; *DC*, decelerative capacity; *EveREst*, Event Rate Estimate; *PlGF*, placental growth factor. *Bowe. Placenta-associated biomarkers and objective cardiotocography patterns. Am J Obstet Gynecol Glob Rep 2022.*

The EveREst plot was used to examine the relationship between PlGF and the rate of DC >6.7 bpm in the “uncomplicated” and “complicated” group (Figure 4). The rate of DC >6.7 bpm for the “uncomplicated” group increases from 4% in the first 0–20th quantile of PlGF level to 8% in the last 80–100th quantile (χ^2^=3.28; *P*=.07; rates in the last quantile group compared with those in the first quantile group). In comparison, the rate of DC >6.7 bpm for the “complicated” group rises sharply from 0% in the first 0–40th quantile groups of PlGF and thereafter a steady rate of 17% in the last 80–100th quantile (χ^2^=1.44; *P*=.23; rates in the last quantile group compared with those in the first quantile groups) (Figure 4).

## Comment

### Principal findings

In this study of predelivery placenta-associated biomarkers and intrapartum computerized FHR patterns, we found that in pregnancies with lower mean maternal predelivery PlGF concentrations, indicative of more stressed placentas, there are more automated OxSys 1.7 alerts compared with those with higher PlGF levels. We also showed increasing rates of DC alerts, indicative of increased risk of fetal acidemia, in the population with lower levels of PlGF relative to the population with higher levels. There was a trend toward a greater increase in the “complicated” group with adverse neonatal outcome compared with the “uncomplicated” group without adverse neonatal outcome.

### Results and clinical implications

We propose that a low proangiogenic predelivery profile (low PlGF) may represent a general marker for syncytiotrophoblast stress.^1^ Low PlGF in maternal circulation at ≥36+0 weeks’ gestation has been shown to be associated with fetal compromise^3^, ^4^, ^5^ and adverse pregnancy outcome in postdate women, as our previous research has shown.^6^ Similarly, DC is associated with fetal compromise and acidemia in labor.^11^ Our hypothesis that increasing placental stress toward the end of pregnancy, as evaluated by placenta-associated biomarkers, would be associated with increased intrapartum fetal stress regardless of outcome group was confirmed by our findings.

Furthermore, our results show that the “complicated” pregnancy group tends to have lower maternal circulating proangiogenic PlGF levels and a higher rate of automated alerts with DC >6.7 bpm compared with the “uncomplicated” group. The lower PlGF and higher rate of alerts show that the “complicated” group had more stressed placentas than the “uncomplicated” group, indicating fetal compromise.

Interestingly, we found no correlation between the maternal predelivery PlGF level and the maximum DC throughout the CTG. The DC measures the frequency and magnitude of FHR decelerations^17^ and has been shown to identify the development of fetal hypotension during labor-like hypoxia in animal models.^16^ However, the DC does not provide direct feedback on how well a certain fetus is adjusting to inflicted stress,^20^^,^^21^ but assesses risk on the basis of threshold values.^16^ The absence of correlation could be because of our sample size, lack of a DC threshold value, and/or placental health being but one aspect of the compensatory stress handling mechanisms.

The observed weak negative correlations between predelivery mean maternal sFlt-1 concentration and STV values (at start and end of trace), and between sFlt-1–PlGF ratio and STV at end of trace suggest that stressed placentas have less capacity to tolerate the additional demands that labor imposes. In contrast to the antenatal negative correlation between STV and chronic hypoxia,^22^^,^^23^ an increase in STV during labor in the early intrapartum stages in cases of acute hypoxia was described in a study by Lu et al.^24^ A total of 1070 deliveries with scalp blood sample taken during labor (because of unsatisfactory FHR trace) were included in the study by Lu et al.^24^ The authors hypothesized that increased levels of catecholamines in lactacidemic fetuses play a role in this STV increase.^24^ Our analyses are not adjusted for multiple testing and should be interpreted accordingly. Therefore, until larger studies add further knowledge, clinical implications of the currently available research, including the work presented herein, should be considered with caution.

### Research implications

Placenta-associated biomarkers are promising predictors of several phenotypes of placental stress.^3^, ^4^, ^5^, ^6^ However, in contrast to the prediction of preeclampsia,^25^ the prediction of fetal compromise cannot be solely biomarker-based. In this study, we applied predelivery maternal biomarkers, taken before labor onset; nevertheless, our findings correspond with previous findings of lower maternal PlGF levels during labor among women with an abnormal CTG pattern.^5^ Furthermore, we chose computerized CTG as an objective method, as opposed to conventional visually assessed CTG, to eliminate the subjectivity of CTG interpretation.

Currently, the threshold of OxSys alerts is adjusted if there is preeclampsia or thick meconium, which has led to clear improvements of sensitivity and specificity.^11^ According to findings reported herein, OxSys 1.7 could be customized to incorporate angiogenic biomarkers, adjusting the threshold on the basis of the biomarkers to potentially improve its accuracy. If this could be performed, it would be of interest to repeat the study and use clinical outcomes directly.

### Strengths and limitations

An important strength of our study is that all neonatal and delivery outcomes were reviewed by an independent clinical expert group, blinded for biomarkers and visual or computerized CTG results, which concluded whether neonatal outcomes were adverse. By applying the umbilical cord blood gas validation as suggested by Kro et al,^15^ we assured a high level of data quality regarding adverse neonatal outcome definition.

Study limitations include a relatively small dataset, low ethnic heterogeneity for external validity, and a high percentage of highly educated women, partly explained by the inclusion criteria (Norwegian and English language). The predictive accuracy of the biomarkers would likely improve with a larger sample size and more adverse neonatal outcomes.

## Conclusions

This study investigated the association of placental health, as evaluated by prelabor maternal circulating placenta-associated biomarkers, and the fetal capacity to tolerate labor stress, as evaluated by objective intrapartum computerized FHR patterns. Our findings support our hypothesis that in pregnancies with low “proangiogenic” PlGF level, indicative of impaired placental function, abnormal FHR patterns (evaluated by OxSys 1.7) during labor are more likely. Therefore, we can expect that computerized electronic fetal monitoring could be improved by adjusting the thresholds of OxSys with respect to the level of placenta-associated biomarkers.

## Appendix Supplementary materials

Image, application 1

Image, application 2
