# Supplementary material for: Predelivery placenta-associated biomarkers and computerized intrapartum fetal heart rate patterns
Source: AJOG Glob Rep. 2022 Dec 16;3(1):100149. doi: 10.1016/j.xagr.2022.100149 (PMC9840179; doi:10.1016/j.xagr.2022.100149)
Supplement: Supplementary file 1 [file mmc1.docx]

**Table S1.** Distribution of the primary adverse neonatal outcome components for the “complicated” group (n=32). In the “complicated” group 21 pregnancies fulfilled one criterion, six fulfilled two, three fulfilled three, one fulfilled four and one fulfilled six criteria.

| **Component** | **N of “complicated group** | **% of “complicated” group^a^** |
| --- | --- | --- |
| Fetal acidemia | 11 | 34% |
| Newborn low Apgar | 21 | 66% |
| Newborn asphyxia | 3 | 9% |
| Intrauterine fetal demise/intra-/post-partum fetal death | 0 | 0 |
| Neonatal intubation/mechanical ventilation >6 hours | 9 | 28% |
| Meconium aspiration syndrome | 4 | 13% |
| Neonatal hypoxic-ischemic encephalopathy | 2 | 6% |
| Therapeutic hypothermia of the neonate | 2 | 6% |

^a^ Percentage of pregnancies of the “complicated” group (n=32) who were diagnosed with each specific adverse outcome.
