# Supplementary material for: Predelivery placenta-associated biomarkers and computerized intrapartum fetal heart rate patterns
Source: AJOG Glob Rep. 2022 Dec 16;3(1):100149. doi: 10.1016/j.xagr.2022.100149 (PMC9840179; doi:10.1016/j.xagr.2022.100149)
Supplement: Supplementary file 2 [file mmc2.docx]

**Table S2.** Correlation between the biomarkers (*PlGF (placental growth factor), sFlt-1 (soluble fms-like tyrosine kinase) and sFlt-1/PlGF ratio)* and the CTG parameters (*Maximum Decelerative capacity (DC), baseline initial, baseline end, Short term variance (STV) at start and STV at end*) in the pregnancy cohort with intrapartum fetal monitoring (CTG cohort) (n=956)

*Significant on a 0.05 level.

r_s_= Spearman’s correlation coefficient.

The CTG cohort, n=956

|  | Maximum Decelerative capacity (DC) | Baseline initial | Baseline end | Short term variance (STV) start | Short term variance (STV) end |
| --- | --- | --- | --- | --- | --- |
| PlGF | r_s_=0.01, p=0.79 | r_s_=0.00, p=0.97 | r_s_=0.02, p=0.62 | r_s_=0.03, p=0.31 | r_s_=0.05, p=0.11 |
| sFlt-1 | r_s_=-0.01, p=0.78 | r_s_=0.03, p=0.44 | r_s_=0.03, p=0.29 | r_s_=-0.07, p=0.04* | r_s_=-0.07, p=0.04* |
| sFlt-1/PlGF ratio | r_s_=-0.01, p=0.75 | r_s_=0.01, p=0.84 | r_s_=0.00, p=0.94 | r_s_=-0.06, p=0.07 | r_s_=-0.07, p=0.03* |
